# d-serine availability modulates prefrontal cortex inhibitory interneuron development and circuit maturation

**DOI:** 10.1038/s41598-023-35615-5

**Published:** 2023-06-13

**Authors:** Oluwarotimi O. Folorunso, Stephanie E. Brown, Jugajyoti Baruah, Theresa L. Harvey, Shekib A. Jami, Inna Radzishevsky, Herman Wolosker, James M. McNally, John A. Gray, Anju Vasudevan, Darrick T. Balu

**Affiliations:** 1 Division of Basic Neuroscience, McLean Hospital, Belmont, MA 02478 USA; 2 Department of Psychiatry, Harvard Medical School, Boston, MA 02215 USA; 3 Angiogenesis and Brain Development Laboratory, Department of Neurosciences, Huntington Medical Research Institutes (HMRI), Pasadena, CA 91105 USA; 4 Center for Neuroscience, University of California Davis, Davis, CA 95616 USA; 5 Department of Biochemistry, Rappaport Faculty of Medicine, Technion-Israel Institute of Technology, Haifa, Israel; 6 VA Boston Healthcare System, West Roxbury, MA 02132 USA

**Keywords:** Molecular neuroscience, Autism spectrum disorders, Schizophrenia

## Abstract

The proper development and function of telencephalic GABAergic interneurons is critical for maintaining the excitation and inhibition (E/I) balance in cortical circuits. Glutamate contributes to cortical interneuron (CIN) development via *N*-methyl-d-aspartate receptors (NMDARs). NMDAR activation requires the binding of a co-agonist, either glycine or d-serine. d-serine (co-agonist at many mature forebrain synapses) is racemized by the neuronal enzyme serine racemase (SR) from l-serine. We utilized constitutive SR knockout (SR^−/−^) mice to investigate the effect of d-serine availability on the development of CINs and inhibitory synapses in the prelimbic cortex (PrL). We found that most immature Lhx6 + CINs expressed SR and the obligatory NMDAR subunit NR1. At embryonic day 15, SR^−/−^ mice had an accumulation of GABA and increased mitotic proliferation in the ganglionic eminence and fewer *Gad1* + (glutamic acid decarboxylase 67 kDa; GAD67) cells in the E18 neocortex. Lhx6 + cells develop into parvalbumin (PV+) and somatostatin (Sst+) CINs. In the PrL of postnatal day (PND) 16 SR^−/−^ mice, there was a significant decrease in GAD67+ and PV+, but not SST + CIN density, which was associated with reduced inhibitory postsynaptic potentials in layer 2/3 pyramidal neurons. These results demonstrate that D-serine availability is essential for prenatal CIN development and postnatal cortical circuit maturation.

## Introduction

Cortical interneurons (CINs) derived from the ventral medial ganglionic eminence (MGE) shape several aspects of cortical circuit maturation during development and maintain cortical excitatory-inhibitory (E/I) balance^1–3^. By maintaining the E/I balance, CINs are critical in promoting efficient information processing and higher cognitive functions^1–3^. The identities and number of CINs relevant for signal processing differ depending on the spatial and temporal control of progenitor cells originating from the MGE. CINs migrating from the MGE mature and ultimately form connections with excitatory pyramidal neurons in the neocortex.

Much of the progress in understanding how the MGE generates CINs subtypes has come from intrinsic genetic programs driven by specific transcription factors including Lhx-6, a LIM homeodomain transcription factor^4–6^. Lhx6 is a master regulator of MGE-derived CINs and hippocampal interneurons (HINs), and is both necessary and sufficient for the tangential migration of most CINs out of the MGE and the differentiation and positioning of these CINs in specific cortical layers^5, 7^. Lhx6 + cells primarily differentiate into parvalbumin (PV) and somatostatin (Sst) interneuron subtypes^5, 8^. Prenatal loss of Lhx6 results in drastically fewer PV+ and Sst+ interneurons in the neocortex and hippocampus^5^. This results in fewer spontaneous inhibitory postsynaptic currents in the dentate gyrus, which leads to decreased inhibition. However, conditional deletion of Lhx6 during adulthood does not affect the number of PV + CINs and has no impact on their morphological and physiological properties^9^.

In addition to transcription factors such as Lhx6, intracellular and extracellular signals also affect interneuron numbers^10^. There is accumulating evidence that activation of ionotropic *N*-methyl-d-aspartate receptors (NMDARs) contribute to CIN development. Before synaptogenesis, NMDARs located on migrating INs, provide a critical source of Ca^2+^ entry^11, 12^. NMDARs on immature and migrating MGE-derived progenitors, regulate the maturation of PV+ and Sst + CINs at juvenile and adolescent time points^13^. NMDARs are unique because they require the binding of a co-agonist, d-serine or glycine to open. d-serine is racemized from l-serine by the neuronal enzyme serine racemase (SR)^14^, and is the primary co-agonist required for synaptic NMDAR activity and NMDAR-dependent plasticity at many mature forebrain synapses^15–17^. Our recent work supports a novel autocrine mode of synaptic d-serine, showing that SR is localized to the postsynaptic but not presynaptic regions of excitatory synapses at glutamatergic and inhibitory cortical neurons^18, 19^.

Here, we investigated whether d-serine regulates CIN development by using a transgenic mouse model lacking SR (SR^−/−^)^14^. First, we show that d-serine levels in embryonic mouse brains are similar to early postnatal time points in mice^20^, with widespread SR and the obligatory NMDAR subunit (GluN1) expression in Lhx6 + cells during embryonic and juvenile development. Compared to WT mice, we find that constitutive SR^−/−^ mice exhibit reduced GABA immunoreactivity and fewer neocortical glutamic acid decarboxylase 67 kDa (GAD67; *Gad1*+) cells in the neocortex during embryonic development. The developmental disruption of CINs in SR^−/−^ mice persists postnatally, as SR^-/-^ mice have fewer GAD67+ and PV + prelimbic (PrL) interneurons than WT mice at postnatal day (PND) 16. We also observe a significant reduction in inhibitory synapses onto layer 2/3 PrL pyramidal neurons in PND16 SR^−/−^ mice, increasing E/I balance. Furthermore, we observed an increase in the intrinsic excitability of layer 2/3 PrL pyramidal neurons. These results provide evidence that the production, distribution, and organization of CINs are disrupted in SR^−/−^ mice, leading to alterations in E/I balance at early postnatal time points.

## Results

### SR is widely expressed in embryonic cortical interneurons (CIN)

**Figure 1:**
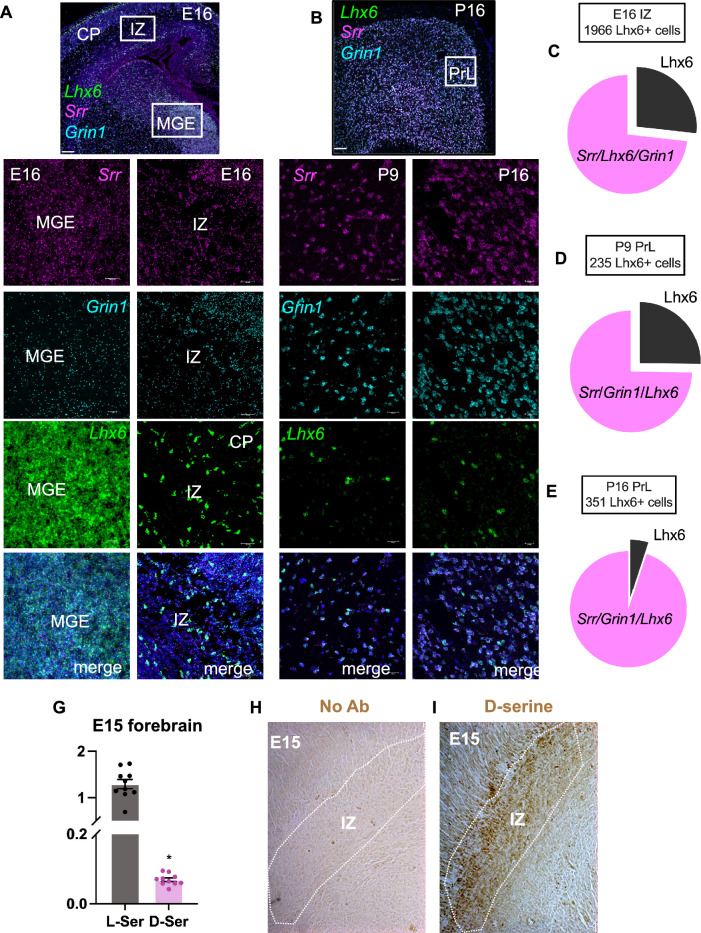
*Srr* expression in Lhx6 + cortical interneuron precursors. (**A**–**C**) mRNA for SR (*Srr*; magenta) and GluN1 (*Grin1*; cyan) are highly expressed in *Lhx6* + intermediate interneuron (green) cells at embryonic day 16 (E16; medial ganglionic eminence (MGE), intermediate zone (IZ), cortical plate (CP)). (**D**) Pie chart represents the percentage of *Lhx6* + cells that express *Srr* and *Srr*/*Grin1* in E16 IZ (n = 3; two sections per animal). (**E**) SR and Grin 1 mRNA expression in postnatal day 9 (PND9) PrL (**F**) Pie chart represents the percentage of Lhx6 + cells expressing *Srr and Grin1* in PND9 and PND16 PrL (n = 2; two sections per animal). (**G**) High-performance liquid chromatography (HPLC) analysis showing l-serine and d-serine content in E15 forebrain tissue (n = 6–10). (**H**) No immunoreactivity when only d-serine antibody is excluded. (**I**) Strong d-serine immunoreactivity (brown) in the IZ migratory stream in E15 forebrain. Scale bar = 100 or 20 µm (H–I) and 10 or 25 µm (A–B). N = Bars represent mean ± SEM.

To determine the effect of d-serine on CIN lineage, we quantified the expression of SR (*Srr*) and the obligatory NMDAR subunit GluN1 (*Grin1*) in cells that express Lhx6 (Fig. 1A–E). We show using multiplex fluorescent in situ hybridization that nearly all *Lhx6* + INs express *Srr* and *Grin1* during prenatal and early postnatal periods (Fig. 1A–B). The outlined cells in the intermediate zone (IZ) at E16 (Fig. 1A) show co-expression of *Lhx6*, *Srr*, and *Grin1* mRNA. Figure 1A,C shows that 89% and 73% of cells at E16 in the IZ are *Lhx6*+/*Srr*+ and *Lhx6*+/*Srr*+/*Grin1*+, respectively. We also observed a temporal increase in the co-expression of *Lhx6*+/*Srr*+/*Grin1*+ *cells* at PND9 (74%) and 16 (95%) in the PrL (Fig 1B,D,E). We show that d-serine and l-serine levels in the E15 forebrain (Fig. 1G) are equivalent to levels observed during the first postnatal week^20^. Furthermore, d-serine is enriched in migrating interneurons of the developing neocortex (Fig. 1H–I).

### SR^−/−^ mice display fewer immature CINs in the embryonic cortex and have an altered density of GABAergic neurons in juvenile PrL cortex

**Figure 2:**
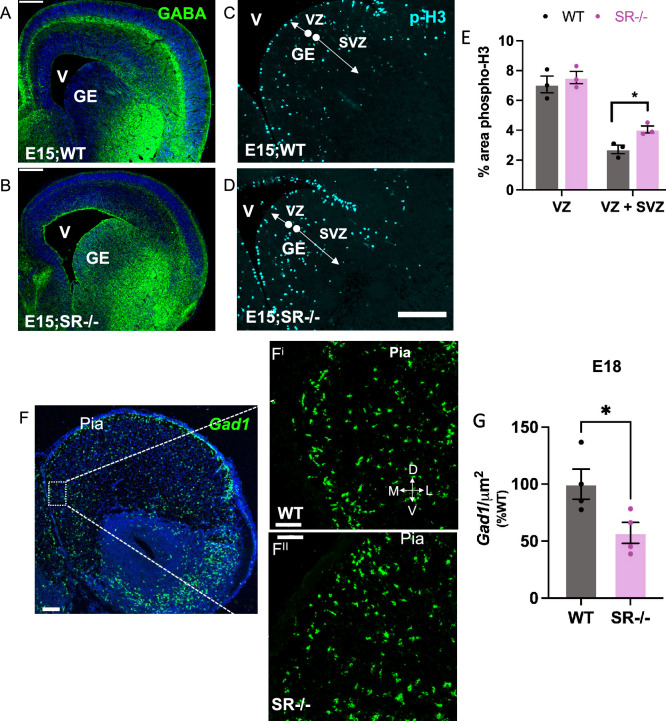
Fewer immature CINs in the embryonic SR^−/−^ cortex. The images show a dramatic reduction in GABA (green) immunofluorescence in the ganglionic eminence (GE), intermediate zone (IZ), and neocortex (white arrows, (**A**,**B**) and increased mitotic phospho-H3 (cyan) expression (**C**,**D**) in SR^−/−^ E15 forebrain compared to WT. (**E**) Quantification of percent area of phospho-H3 positive cells in the ventricular zone (VZ) and subventricular zone (SVZ) within the GE. (n = 3–4). Scale bar—50 µM. (**F**) In situ hybridization showing *Gad1 mRNA* expression in WT (**F**^**i**^) and SR^−/−^ (**F**^**ii**^) E18 neocortex. (WT-black, SR^−/−^-magenta). Data represent means ± SEM. Unpaired t test *p < 0.05. V—ventricle. Scale bar—50 µM (**A**,**B**) 100 µM; and 50 µM (**F**) 100 µM; **F**^**i & ii**^—50 µM.

**Figure 3:**
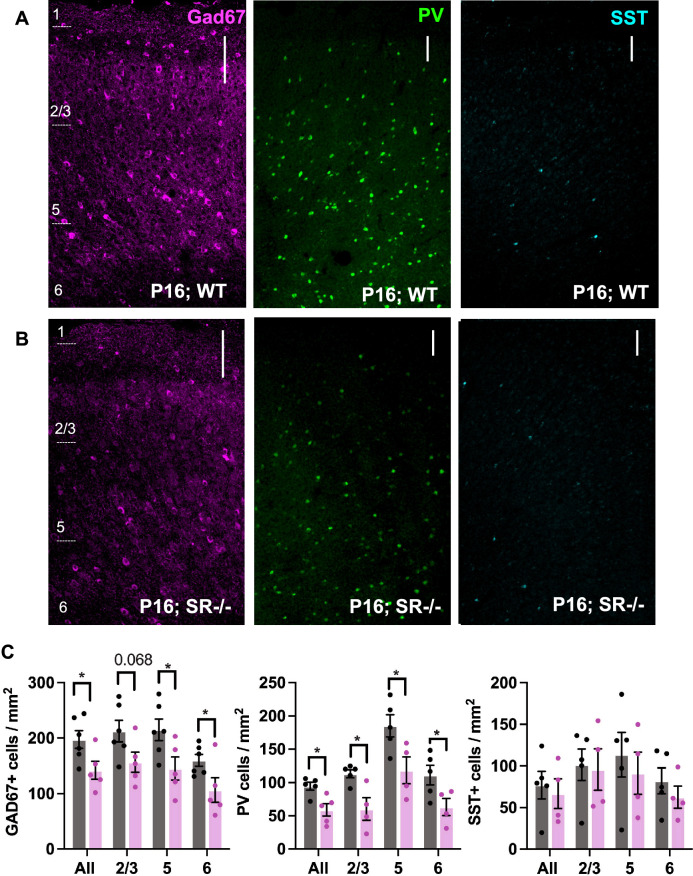
GAD67+ and PV+ cell densities are reduced in the PrL of juvenile SR^−/−^ mice. Glutamic acid decarboxylase (Gad67; magenta), Parvalbumin (PV; green), somatostatin (Sst; cyan) immunostaining from PND16 PrL. (**A**) WT, (**B**) SR^−/−^ (**C**) Quantification of Gad67+, PV+, SST+ cell densities in WT (black) and SR^−/−^ (magenta) PND16 PrL cortical layers. Data represent means ± SEM. (n = 4–6). Unpaired student′s t test. *p < 0.05 (Scale bar—100 µM).

Given the widespread expression of SR and GluN1 in Lhx6+ cells embryonically, we next sought to determine if genetically eliminating SR and thus reducing d-serine would alter IN development and the levels of GABAergic neurons in juvenile mice. Since the cortical interneuron progenitor cells are derived from the ventral ganglionic eminence, we used GABA and phospho-histone3 (p-H3; postmitotic marker) immunoreactivity to determine if there are changes in the distribution of progenitors in the GE. Compared to WT, SR^−/−^ forebrains exhibit profound accumulation of GABA (Fig. 2A,B) and a significant increase in p-H3 in the ganglionic eminence at E15 (t^4^ = 3.63, p = 0.022; Fig. 2C–E). GABA immunoreactivity at E15 in the IZ (Fig. 2A,B), and *Gad1* mRNA at E18 (Fig. 2F) in the frontal cortex, were used to visualize immature INs in WT and SR^−/−^ neocortex. We show markedly reduced GABA immunoreactivity in the neocortex of SR^−/−^ mice (Fig. 2A,B). We further found that the number of *Gad1*+ cells in SR^−/−^ cortex at E18 was reduced by 20% compared to WT mice (t_6_ = 2.67, p = 0.037; Fig. 2G). While functional impairment of GABAergic CINs has been proposed to be one of the major factors contributing to E/I imbalance in various psychiatric disorders, most of the attention has been directed towards PV + GABAergic INs dysfunction. At PND16, we found a ~ 30% reduction of both GAD67+ and PV+ cells in the PrL of SR^−/−^ mice across all cortical layers (total Gad67: t_9_ = 2.43, p = 0.038; PV: t_8_ = 3.23, p = 0.012; Fig. 3A–C, Table 1). There was no significant reduction in the number of Sst+ cells in the PrL of SR^−/−^ mice (total SST t_7_ = 0.42, p = 0.687, Fig. 3A–C, Table 1). Given that half of all hippocampal neurons originate from the MGE, we also observed a significant reduction in Gad67+, PV+ and SST+ cells in the hippocampus (Supplementary Fig. 1). In both the PrL and hippocampus (Supplementary Fig. 2A,B), we found no notable variation in PV + density between the SR^−/−^ and WT at postnatal day 29.

**Table 1:** Student t-test showing interneuron densities changes in P16 PrL cortical layers in wild-type versus SR^−/−^.

PrL layers	GAD67	PV	Sst
Layer 2/3	p = 0.068 t(9) = 2.07	p = 0.014 t(7) = 3.25	p = 0.857 t(7) = 0.19
Layer 5	p = 0.037 t(7) = 2.44	p = 0.036 t(7) = 2.59	p = 0.572 t(7) = 0.59
Layer 6	p = 0.048 t(9) = 2.29	p = 0.049 t(7) = 2.38	p = 0.387 t(7) = 0.92

### Increased E/I ratio in layer 2/3 PrL pyramidal neurons of SR^−/−^ mice

**Figure 4:**
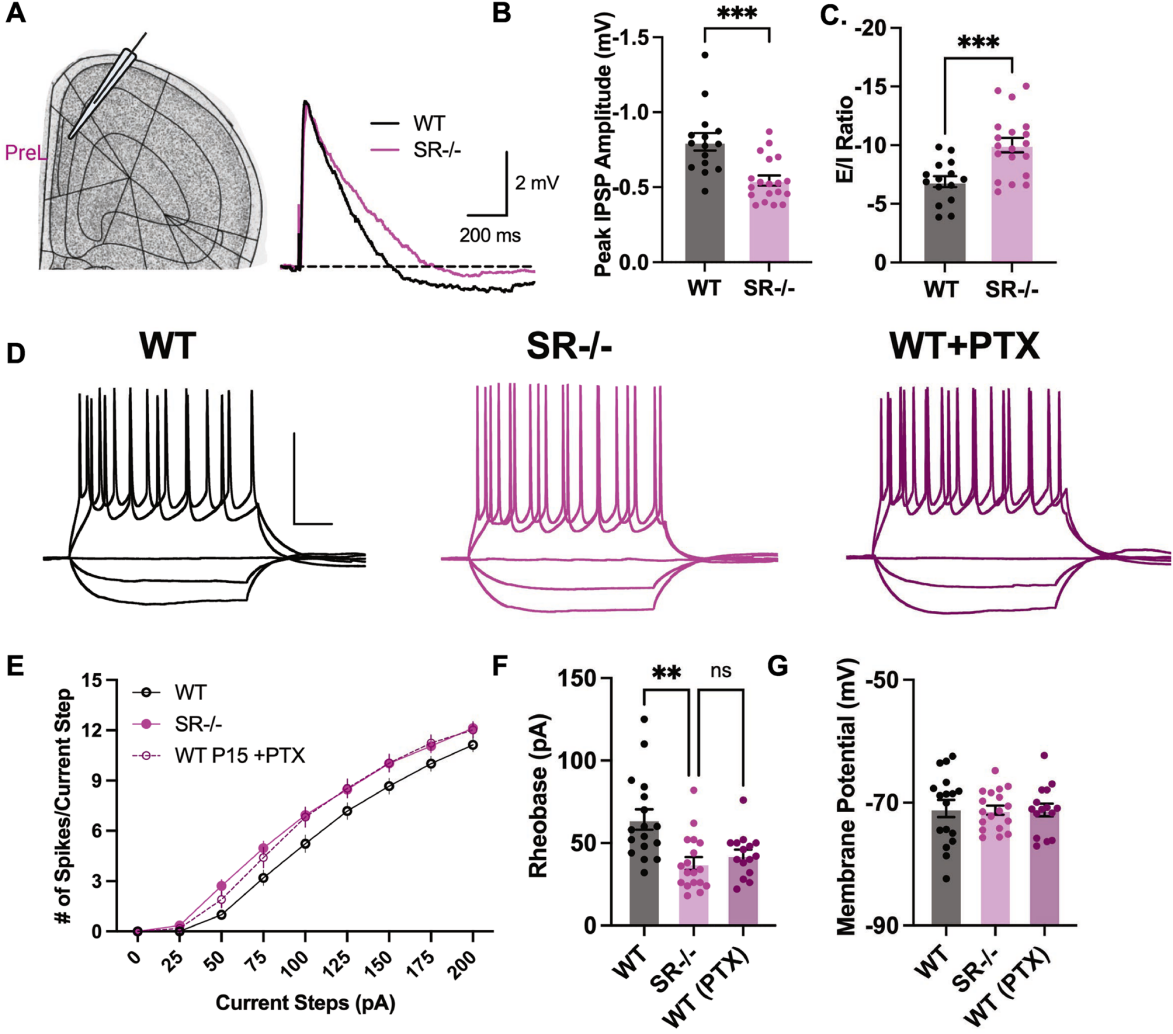
Increased excitation/inhibition (E/I) ratio and enhanced pyramidal neurons intrinsic excitability in juvenile SR^−/−^ PrL *layer* 2/3 pyramidal neurons. (**A**) The Images show PrL *layer* 2/3 that was recorded and the compound postsynaptic potentials (PSP) from PrL*layer* 2/3 pyramidal neurons evoked by superficial *layer* 5 pyramidal stimulation in the absence of synaptic blockers at a holding potential of − 60 mV. Dashed line indicates the baseline; scale bars: 2 mV, 200 ms. Bar graphs showing (**B**). E/I ratio calculated by the peak amplitudes of depolarization ("EPSP") and hyperpolarization ("IPSP"). (**C**) Peak hyperpolarization ("IPSP") amplitude in the 600 ms after the stimulus. (n = 4; 15–19 cells/genotype; WT—black, and SR^−/−^—magenta). (**D**) Sample traces for 0, − 100, − 200, + 100, and + 200 pA current steps for WT, SR^−/−^ and WT plus picrotoxin (GABA_A_ receptor antagonist). Scale bars: 50 mV, 100 ms. (**E**) Depolarization induced by somatic current injection elicits an increase in the numbers of APs in the SR^−/−^ compared to WT cells (n = 4; 15–19 cells/genotype). Summary graph of (**F**) Rheobase and (**G**) Resting membrane potential (Rm) (n = 4 mice; 15–19 cells/genotype; WT—black, SR^−/−^—magenta, WT plus picrotoxin—purple). Data represent means ± SEM. Unpaired student's t-test. *p < 0.05. ***p < 0.001.

Since SR^−/−^ mice had fewer PV + CINs in the PrL, we investigated the properties of excitatory synaptic transmission in *layer* 2/3 PrL of WT and SR^−/−^ mice (Fig. 4A). We recorded compound PSPs using whole-cell current clamps at a holding potential of − 60 mV with current injection upon superficial *layer* 5 stimulation. For this experiment, the peak depolarization of the PSP was set to approximately 5 mV (WT: 5.20 ± 0.18 mV, n = 15; SR^−/−^: 5.11 ± 0.08, n = 19, t_32_ = 0.52, p = 0.605) to draw out the inhibitory component of compound PSPs (Fig. 4A). We found a significant reduction in the IPSP component of the compound EPSP/IPSP (Fig. 4B, peak IPSP amplitude, t_32_ = 4.04 p < 0.001). The decrease in IPSP amplitude resulted in an increased E/I ratio (Fig. 4C; E/I ratio, t_32_ = 3.84, p < 0.001). These results suggest that a selective GABAergic impairment in SR^−/−^ mice leads to an increase in the E/I balance.

### Increased intrinsic excitability in SR^−/−^ PrL pyramidal neurons

Next, we analyzed the number of spikes elicited during 500 ms steps of somatically injected current and found a significant increase in the intrinsic excitability of layer 2/3 PrL pyramidal cells in SR^−/−^ mice compared to WT mice (Fig. 4D,E). This increased intrinsic excitability was associated with a significant decrease in the rheobase (WT: 64.3 ± 6.2 pA, *n* = 17; SR^−/−^: 37.7 ± 3.9 pA, *n* = 18, t^33^ = 3.68, p < 0.001), but did not affect the resting membrane potential or the action potential threshold (Fig. 4F,G, Table 2). The magnitude of the increased intrinsic excitability SR^−/−^ mice was mimicked in WT mice by adding the GABA_A_ receptor antagonist picrotoxin (Fig. 4F), suggesting reduced tonic inhibition contributed to the increased firing of SR^−/−^ neurons. Together, these data suggest that a reduction in inhibitory input onto *layer* 2/3 PrL pyramidal neurons in SR^−/−^ mice increases the E/I balance resulting in enhanced synaptically driven neuronal excitability.

**Table 2:** Intrinsic excitability in P15 wild-type versus SR^−/−^ layer 2/3 pyramidal neurons.

Property	WT (n = 17)	SRKO (n = 18)	Student's t-test (unpaired)	p value
RMP (mV)	− 71.0 ± 1.4 [− 82.4 to − 62.5]	− 71.2 ± 0.8 [− 75.7 to − 64.8]	t(33) = 0.171	0.865
R_input_(MΩ)^a^	302.3 ± 17.9 [158.9 to 439.3]	308.4 ± 15.4 [158.6 to 427.9]	t(33) = 0.259	0.798
Rheobase (pA)	64.3 ± 6.2 [32.0 to 125.0]	37.7 ± 3.9 [18.0 to 82.0]	t(33) = 3.68	0.0008***
AP threshold (mV)^b^	− 54.2 ± 2.0 [− 64.1 to − 38.4]	− 52.9 ± 1.2 [− 65.4 to − 44.8]	t(33) = 0.581	0.565
AP height (mV)	115.0 ± 2.6 [101 to 140]	118.2 ± 2.0 [106 to 135]	t(33) = 0.984	0.333

Mean ± SEM [range].

^a^Rinput calculated at − 100 pA injection.

^b^Junction potential not adjusted.

## Discussion

The results presented in this study show that immature Lhx6 + CINs originating from the MGE robustly express *Grin1* and *Srr* mRNA. We show that d-serine is critical for (1) the proper distribution of CINs during embryonic development and, (2) maintaining the proper density of PV + CINs in the juvenile PrL and hippocampus. This CIN deficiency in PND16 SR^−/−^ mice was associated with disrupted pyramidal neuron inhibition and increased firing of layer 2/3 of the PrL pyramidal neurons. Overall, our data suggest that SR, which is highly expressed in CINs at the investigated time points, is important for regulating CIN development, maturation, and prefrontal (E/I) balance.

Our findings in embryonic SR^−/−^ mice are consistent with previous studies suggesting that NMDAR activation is important for maintaining MGE-derived IN production, migration, morphology, and synaptic connectivity. For example, treatment of E16 rat brain slices with NMDA, in the absence of Mg^2+^, increased Ca^2+^ influx into migrating CINs in the IZ^12^. NMDARs are expressed and become active at E15^21, 22^, which coincides with the second and more prominent stream of INs, mainly from MGE that rapidly migrate into the neocortex through the IZ^23^. We show that d-serine is also expressed in the IZ at E15, and that d-serine elimination causes a reduction of GABA in the migratory stream (Fig. 2A–B). We observed an increase in proliferation in the eminence of SR^−/−^ mice which is similar to results reported in cell models with either genetic mutations of Grin2B or administration of a competitive NMDAR antagonist^24^. Reducing Grin2B mRNA by 50% or treating with (2-amino-5-phosphonovalerate (APV; NMDAR antagonist) and ifenprodil (GluN2B antagonist), increased the proliferation of human neural progenitor cells^24^. Finally, d-serine produced by primary cultured NSCs supports its proliferation and neuronal differentiation^25^. Altogether, these studies suggest that constitutive SR elimination resembles embryonic NMDAR hypofunction and disrupts prenatal IN development.

We show here that d-serine reduction causes a 30% reduction of PV + CIN density, increases E/I ratio, and firing of PrL pyramidal neurons at PND16 (Fig. 3), which supports the hypothesis that PV + CINs are essential for maintaining the E/I ratio. Our findings are also supported by studies that show that activation of PV + CINs in the mPFC rescues E/I imbalance and social deficits^2^. Furthermore, a 25% reduction of adult PV CIN density in the PFC was sufficient to reduce local GABAergic transmission onto pyramidal neurons, disrupt prefrontal E/I balance, and alter the processing of afferent information from the ventral hippocampus^26^. Although we did not observe  any changes in PV+ cell density at PND29 (Supplementary Fig. 2A), we cannot rule out changes in PV function. Previous studies have shown that selective knockdown of the NMDAR subunit GluN1 in the mPFC PV+ interneuron decreases its intrinsic excitability^27^. Furthermore, adult SR knockout mice showed no changes in PV density in the prelimbic cortex^28^. However, these mice showed a decreased preference for social novelty and reduced social investigation-gamma oscillation in the frontal cortex, which PV controls^29^. Together, our data suggest that there might be a delay in the maturation of these PV+ cells in the SR^−/−^ PrL.

NMDAR hypofunction has been postulated to be central to the pathophysiology of neurodevelopmental disorders that share cognitive and social deficits^30^. However, the timing and cell-type specificity of NMDAR hypofunction needs further investigation. Conditional genetic elimination of GluN1 on Nkx2.1 + cells (MGE-Grin1^fl/fl^) in the MGE causes widespread downregulation of synaptic transcriptional programs in PV+ and Sst + CIN subtypes at PND20 and reduces the number of PV+ neurons in the frontal cortex at PND30^13^. Furthermore, NMDAR activation during PND7-9 is essential for maintaining inhibitory synapse density at PND7 and synaptic activity of PND21 CINs^31^. Interestingly, post-adolescent deletion of Grin1 did not affect IN density, firing rate, or synchronous firing of cortical excitatory neurons, suggesting that early postnatal inhibition of NMDAR activity on INs is essential for the development of pathology^32^. In a study involving a mouse model of autism spectrum disorder (neuroligin 3 R451C knockin mice), d-Cycloserine (DCS, a partial agonist that binds at the same site as D-serine) was found to improve PV dysfunction^27^. Additionally, direct infusion of DCS into the mPFC during adolescence resulted in rescued deficits in social novelty preference in adults^27^. Further research is needed to establish the impact of administering d-serine during prenatal and early postnatal stages on the PV + cell density. Lastly, our findings are consistent with the effect of GABAA receptor antagonists on pyramidal neurons (Fig. 4) and suggest that d-serine is vital for PV development and activity in the PrL.

We hypothesize that D-serine availability during development may impact the PV and SST neuronal groups differently, and the region-specific diversity may explain the selective vulnerability of the PV+ population we observe in the cortex^33^. Firstly, the generation of SST + neurons takes place in the dorsal MGE and reaches its peak at E14.5. On the other hand, PV + neurons originate in the ventral MGE and attain their maximum only at E15.5^33^. We predict that we might observe changes in SST + neurons at earlier time points in the prelimbic cortex. Secondly, PV + interneurons comprise around 40–50% of the GABAergic interneuron population in the neocortex, while SST + interneurons contribute 30% of GABAergic interneurons. On the other hand, in the hippocampus, PV + interneurons represent 30% of the population, while SST + interneurons represent 50% of GABAergic interneurons^33^. Finally, there might be distinct expression of NMDAR subunits in PV vs. SST neuronal populations that are differentially affected by d-serine availability at these times^34^. It would be interesting to evaluate whether there are alterations in other interneuron populations such as calbindin+, calretinin+, neuropeptide Y (NPY)+, vasoactive intestinal peptide (VIP)+ and cholecystokinin (CCK)+ interneurons that may serve as compensatory mechanisms in the SR^−/−^ PrL.

Studies suggest that NMDAR signaling regulates the MGE-derived IN subtypes in part by innate genetic programs such as, Lhx6 transcription factor. Lhx6 gives rise to PV and SST cells that populate the PFC and hippocampus. Our results are supported by scRNAseq and Ribotag-seq data from MGE-Grin1^fl/fl^ mice that show a significant reduction in the expression and translation of Lhx6 mRNA and its downstream targets in hippocampal IN subtypes and not cortex^13^. In this study, there were differences in the PV and SST subtype clusters in the cortex compared to the hippocampus at PND20. Interestingly, adult knockdown of Lhx6 in the hippocampus did not affect PV density and firing, suggesting that Lhx6 activity during development is essential for IN subtype expression and function^9^. Although we did not observe any changes in Lhx6 mRNA in E17 SR^−/−^ forebrain (data not shown), we cannot rule out MGE-specific changes in expression and activity or any changes in postnatal expression of Lhx6. Future work is needed to uncover how d-serine and NMDAR signaling regulates the abundance and diversity within PV/SST subclasses and other IN subtypes and to identify the signaling pathways downstream of d-serine and NMDAR activation.

Overall, our study demonstrates that d-serine availability is essential for interneuron number in the neocortex during prenatal development and proper circuit maturation in the PrL. Further, we show that SR deficiency results in GABAergic dysfunction through a loss of GABAergic synapses. Future studies will also aim to determine the specific impact of SR deletion in CIN progenitor cells on interneuron density, inhibitory synapses and  E/I balance. The clinical implications of these findings are that NMDAR hypofunction due to reduced co-agonist availability may contribute to the pathophysiology of neurodevelopmental disorders characterized by IN dysfunction.

## Materials and methods

### Experimental design

#### Mice breeding

We used C57BL6/J wild-type (Jackson Lab) and heterozygous (SR^+/−^^14^; for breeding in all experiments. For embryonic experiments, SR^+/−^ female mice were timed mated with either SR^+/−^ or WT males. Timed mating was initiated in the evening, and female mice were checked for vaginal plugs the following morning. Embryonic day 0 (E0) was the first day a female presented with a vaginal plug. Pregnant female mice were group-housed until E14, then single-housed until embryo extraction. For postnatal experiments, SR^+/−^ females were left with SR^+/−^ or WT males for 1 week after initial pairing, group-housed with other pregnant dams until 2 weeks after initial pairing, and then single-housed until the time of pup collection. Mice were maintained in polycarbonate cages on a 12-h light/dark cycle and given access to food and water ad libitum. All procedures were performed in accordance with the National Institutes of Health guidelines, ARRIVE guidelines, and in compliance with the animal protocols approved by The McLean Hospital Institutional Animal Care and Use Committee, and the Institutional Care and Use Committee at the University of California, Davis,

#### Embryonic brains

Embryonic brains extracted at either E15 or E18. Dams were anesthetized with an intraperitoneal injection of Ketamine (100 mg/kg)/Xylazine (5 mg/kg) before extraction. Embryonic brains were fixed in 4% paraformaldehyde (Electron Microscopy Sciences, cat# 19202) overnight, transferred to 20% sucrose until brains sunk to the bottom, and then 30% sucrose. Brains were then frozen on dry ice and stored at – 80 °C. All embryos were genotyped, and both male and female brains were used for embryonic experiments. Embryonic cryostat sections (14 and 20 μm) were directly adhered on glass slides and stored at – 80 °C.

#### Postnatal brains

At postnatal day (PND) 9 and 16, male mice were deeply anesthetized with isoflurane administered in a small chamber, then briefly intracardially perfused with cold 1 × PBS (0.5 M PB, NaCl, pH 7.4), followed by 4% paraformaldehyde (Electron Microscopy Sciences, cat# 19202), and then cryoprotected in 30% sucrose/PBS at 4 °C. For in situ hybridization, brains were sectioned at 14 μm and directly adhered on glass slides that were stored at – 80 °C. For immunofluorescent staining, sections were cut at 30 μm using a Leica SM 2010R Microtome and floating brain sections were stored in a cryoprotectant solution (ethylene glycol, glycerol, 0.5 M PB, NaCl, KCl, in dH20) at − 20 °C.

### Fluorescent in situ hybridization (RNAscope)

14 µm thick sections from embryonic day (16 and 18) and postnatal day (9 and 16) from WT or SR^−/−^ mouse brains were used for RNAscope in situ hybridization. Only male brains were used for postnatal experiments. RNAscope was performed using the RNA Scope Fluorescent Multiplex 2.5 labeling kit (ACD Bio, cat# PN323110) according to the manufacturer's specifications (ACD Bioscience). The following probes were used: mm-Srr-01-c2 (cat# 486271-c2), mm-Grin1-c3 (cat# 431611), mm-Lhx6-c1 (cat# 422791), mm-Gad1 (cat# 400951). The following opal fluorophores were used for visualization: 520 (cat# FP1487001KT), 570 (cat# FP1488001KT), and 690 (cat# FP1497001KT). Z-stacks images obtained with a Leica SP8 confocal microscope were exported and analyzed using HALO imaging analysis software (ISH Quantification Module; indica labs) to quantify *Lhx6*+, *Grin1*+, *Srr*+. For E15, we utilized plate 5, and for E18, we used plate 3^35^.

### Immunofluorescence staining

Brain sections were washed three times in 1 × PBS, incubated in blocking buffer (10% goat serum, 1% BSA and 0.1% triton in PBS) for an hour, and then incubated overnight with primary antibodies in blocking buffer at 4 °C. The primary antibodies used were rabbit anti-GABA (cat# A2052; 1:100), rabbit anti-phospho-H3 (cat# 06-570; 1:150), guinea pig anti-parvalbumin (cat# 195004; 1:500), guinea pig anti-somatostatin (cat# 366004;1:300), and mouse anti-GAD67 (cat# MAB5406; 1:1000). Sections were incubated in appropriate secondary antibody in blocking buffer for an hour at room temperature. The secondary antibodies used were anti-mouse Alexa 488 conjugate, anti-rabbit Alexa 488 conjugate, anti-guinea pig Alexa 488, anti-goat Alexa 647 conjugate. Sections were washed three times with 1 × PBS, incubated in Hoechst (cat# 3570), washed three times with 1 × PBS, mounted, and cover slipped using ProLong^®^ Gold anti-fade medium. We utilized plate 3 for our experiments^35^.

### d-serine immunohistochemistry

Embryonic forebrains were fixed in solution containing 3% glutaraldehyde (cat#111-30-8, Electron Microscopy Sciences, Hatfield, PA), 1% paraformaldehyde (PFA, cat# 76240, Millipore Sigma), 0.2% sodium metabisulfite (cat# S9000, Millipore Sigma) and 10 U/mL Heparin salt (cat# H3393, Millipore Sigma) overnight. Fixed brains were cryoprotected in sucrose and frozen on powdered dry ice. Brains were sectioned (20 mM) and directly adhered to a glass slide. Sections were washed three times in 0.1 M PBS for 10 min, incubated in 0.5% NaBH4 and 0.2% sodium metabisulfite in TBS (pH 7.4) for 10 min, then washed three times with 1 × TBS for 10 min. Next, sections were incubated in 0.3% H_2_O_2_ in TBS (pH7.4) for 30 min, washed thee times with 1 × TBS for 10 min, then incubated in a blocking solution consisting of TBS (pH7.4), 10% normal goat serum and 0.1% Triton X-100 for 60 min. Sections were then incubated in rabbit anti-d-serine (cat# ab6472 1:30 000) at 4 °C for two nights in a blocking solution containing 5 mM l-serine-BSA-glutaraldehyde conjugate. After two nights, the sections were washed three times with 1× TBS, followed by incubation in goat anti-Rb IMMPRESS HRP reagent (cat# MP7451, Vector, Burlingame, CA) for 60 min at room temperature, washed three times with 1× TBS and revealed by incubating with IMMPACT DAB (cat# SK4105, Vector) for 10 min. Finally, sections were washed with X TBS three times and incubated in increasing ethanol solution (50%, 70%, 95%, 100%, 100%) followed by two 10 min xylene incubations.

### HPLC

E15 forebrains were weighed, 10 volumes of 8% trichloroacetic acid (TCA; T0699, Sigma) was immediately added, and the samples stored at – 80 °C. Brains were homogenized and the supernatant was extracted five times with water-saturated ether to remove TCA and then diluted into 20 mM borate buffer at pH 9.0. The samples were derivatized for 50 s with *N*-tert-butyloxycarbonyl-l-cysteine and o-phthaldialdehyde (MilliporeSigma) using 100 pmol L-2-aminoadipic acid (Millipore Sigma) as an internal standard. Samples were separated using two coupled Chromolith RP-18 endcapped 100-4.6 HPLC columns (Merck, Kenilworth, NJ) in a Hitachi HPLC apparatus (Tokyo, Japan) consisting of a pump (L-7100), autosampler (L-7250), fluorescent detector (L-7485), and degasser (L-7614).

### PV brain-wide imaging and analysis

#### Sample processing and imaging

Mice were perfused transcardially with ice-cold 1× PBS with 10 U/mL heparin until fluid runs clear, followed by ice-cold 4% PFA. Extracted brains were postfixed in 4% PFA solution at 4 °C for 24 h. with gentle shaking. Brains were washed twice in PBS and then store samples in PBS with 0.02% sodium azide before shipping to LifeCanvas Technologies (MA, USA) for subsequent sample processing and imaging. Brains were preserved with SHIELD reagent (LifeCanvas Technologies)^36^. Cleared brains were immunolabeled in SmartLabel (LifeCanvas Technologies, MA, USA) for 24 h using the following primary antibodies that against goat anti-PV (ab32895). Fluorescently conjugated secondary antibodies against appropriate species were applied.^36^ After immunolabeling, samples were incubated in 50% EasyIndex (RI = 1.52, LifeCanvas Technologies) overnight at 37 C followed by 1 day incubation in 100% EasyIndex for refractive index matching. After index matching the samples were imaged using a SmartSPIM axially-swept light sheet microscope using a 3.6x (0.2 NA) (LifeCanvas Technologies). Samples were registered to the Allen Brain Atlas (Allen Institute: https://portal.brain-map.org/) using an automated process (alignment performed by LifeCanvas Technologies). A NeuN channel for each brain was registered to an average NeuN atlas (generated by LCT using previously-registered samples). Registration was performed using successive rigid, affine, and b-spline warping algorithms (SimpleElastix: https://simpleelastix.github.io/). Automated cell detection was performed by LifeCanvas Technologies using a custom convolutional neural network created with the Tensorflow python package (Google). The cell detection was performed by two networks in sequence. First, a fully-convolutional detection network (https://arxiv.org/abs/1605.06211v1) based on a U-Net architecture (https://arxiv.org/abs/1505.04597v1) was used to find possible positive locations. Second, a convolutional network using a ResNet architecture (https://arxiv.org/abs/1512.03385v1) was used to classify each location as positive or negative. Using the previously-calculated Atlas Registration, each cell location was projected onto the Allen Brain Atlas in order to count the number of cells for each atlas-defined region.

### Quantification of CIN densities in PrL

The number of PV, Sst, and GAD67 neurons in PND16 WT and SR^−/−^ PrL were quantified using manual counts by a trained researcher blinded to genotype using the StereoInvestigator software (MBF Bioscience). At least four sections were analyzed from each mouse (*n* = 4–6; section interval = 6) matched from rostral to caudal positions and spanning approximately − 2.95 mm through 1.42 mm bregma (anterior/posterior). The cells were counted at 20 × using exposure and image settings that were consistent across all sections. Density was calculated by dividing the number of counted cells by the total area counted.

### Electrophysiology

#### Slice preparation

Male SR^fl^ (labeled as WT) and SR^−/−^ mice (P15) were deeply anesthetized with isoflurane, followed by cervical dislocation and decapitation^37^. The brain was rapidly removed and submerged in ice-cold, oxygenated (95% O_2_/5% CO_2_) ACSF containing (in mm) as follows: 124 NaCl, 2.4 KCl, 25 NaHCO_3_, 1 NaH_2_PO_4_, 2 CaCl_2_, 1.2 MgSO_4_, and 10 glucose (Sigma-Aldrich). Brains were rapidly removed and 350 µm coronal slices from the mPFC were cut on a Leica VT1200 vibratome (Buffalo Grove, IL) in ice-cold, oxygenated (95% O_2_/5% CO_2_) ACSF. Slices were incubated (at 32 °C) for 20 min and then maintained in submerged-type chambers that were continuously perfused (2–3 ml/min) with oxygenated (95% O_2_/5% CO_2_) ACSF at room temperature and allowed to recover for at least 1.5–2 h before recordings. Just prior to the start of experiments, slices were transferred to a submersion chamber on an upright Olympus microscope, perfused with warmed to 30.4 °C using a temperature controller (Medical System Corp.) normal ACSF saturated with 95% O_2_/5% CO_2._

#### Whole-cell current clamp recordings

*Layer* 2/3 pyramidal neurons were visualized by infrared differential interference contrast microscopy, and current clamp recordings were performed using borosilicate recording electrodes (3–5 MΩ) filled with a K^+^-based electrode-filling solution containing (in mM)-135 K-gluconate, 5 NaCl, 10 HEPES, 2 MgCl_2_, 0.2 EGTA, 10 Na_2_-phosphocreatine, 4 Na-ATP, 0.4 Na-GTP (pH = 7.3, 290 mOsm). To measure evoked postsynaptic potentials (PSPs), a bipolar, nichrome wire stimulating electrode (MicroProbes) was placed superficially to where *Layer* 5 appears and current clamp recordings at holding potential of − 60 mV were made from *Layer* 2/3 pyramidal neurons in the absence of synaptic blockers. E/I ratio was calculated from averaged baseline subtracted traces as the maximum depolarization amplitude (in mV) divided by the maximum hyperpolarization amplitude in the 600 ms after the stimulus.

### Statistical methods

Statistical analyses were performed using Prism 9 (GraphPad Software, San Diego, CA, USA). We used unpaired t-tests to compare genotypes for the neocortex and prelimbic cortex density and E/I. The E/I ratio in PrL pyramidal cells calculated from EPSP and IPSP peak amplitudes was compared using Student's t-tests. Data are presented as mean ± SEM. *p < 0.05, **p < 0.01, ***p < 0.001.

## Supplementary Information

Supplementary Information.

## Data Availability

All data generated or analyzed during this study are included in this published article.
