# Supplementary material for: d-serine availability modulates prefrontal cortex inhibitory interneuron development and circuit maturation
Source: Sci Rep. 2023 Jun 13;13:9595. doi: 10.1038/s41598-023-35615-5 (PMC10264435; doi:10.1038/s41598-023-35615-5)
Supplement: Supplementary file 1 — Supplementary Information. [file 41598_2023_35615_MOESM1_ESM.docx]

**D-serine availability modulates prefrontal cortex inhibitory interneuron development and circuit maturation**

*Oluwarotimi O. Folorunso^1^, Stephanie E. Brown^1^, Jugajyoti Baruah^1,2^, Theresa L. Harvey^1^, Shekib A. Jami^4^, Inna Radzishevsky^3^, Herman Wolosker^3^, James M. McNally^5^, John A. Gray^4^, Anju Vasudevan^2^, Darrick T. Balu^1^

*^1^ Division of Basic Neuroscience, McLean Hospital, Belmont, MA 02478, USA.*

*^2^ Angiogenesis and Brain Development Laboratory, Department of Neurosciences, Huntington Medical Research Institutes (HMRI), Pasadena, CA 91105 USA.*

*^3^ Department of Biochemistry, Rappaport Faculty of Medicine, Technion-Israel Institute of Technology, Haifa, Israel.*

*^4^ Center for Neuroscience, University of California Davis, Davis, CA 95616, USA.*

*^5^ VA Boston Healthcare System, West Roxbury, MA 02132, USA.*

**Supplementary Figure 1: PV + cell densities are reduced in the hippocampus of juvenile SR-/- mice.** Immunostaining from PND 16 hippocampus from WT and SR-/-, **A**. Parvalbumin (PV; green), **B**. Glutamic acid decarboxylase (Gad67; magenta), **C**. somatostatin (SST; cyan) **B, D, F.** Quantification of PV+, Gad67+, SST+ cell densities in WT (gray) and SR-/- (magenta) P16 hippocampus. Data represent means ± SEM. (N= 4-6). Unpaired t test. *p<0.05. ***p<0.001 (Scale bar – 100µM).

**Supplementary Figure 2: No change in the number of PV+ cells in adolescent SR -/- mice.** Whole brain immunostaining from PND 29 WT (gray) and SR-/- (magenta) showing the number of PV+ cells in . **A**. prelimbic cortex (PrL). **B**. Hippocampus (HP). DG-dentate gyrus, ML-molecular layer, PL – polymorphic layer, GCL -granule cell layer. Data represent means ± SEM. (N= 4-6). Unpaired t test. *p<0.05.
